# Short Video Addiction on the Interaction of Creative Self-Efficacy and Career Interest to Innovative Design Profession Students

**DOI:** 10.3390/healthcare11040579

**Published:** 2023-02-15

**Authors:** I-Tung Lin, Yu-Min Shen, Mei-Jen Shih, Chien-Chang Ho

**Affiliations:** 1 College of General Education, Chihlee University of Technology, New Taipei City 22050, Taiwan; 2 Department of Industrial Education, National Taiwan Normal University, Taipei City 106, Taiwan; 3 Department of Applied English, Chihlee University of Technology, New Taipei City 22050, Taiwan; 4 Department of Physical Education, Fu Jen Catholic University, New Taipei City 24205, Taiwan

**Keywords:** career interests, creative self-efficacy, short video addiction, innovative design, profession students

## Abstract

In recent years, a variety of emerging online media, such as TikTok, Kuaishou, YouTube and other short video application platforms, have appeared. The problem of short video addiction has become an issue to education experts and the general public, as students’ excessive use of short video has become increasingly serious with many hidden concerns to the students’ learning effectiveness. In addition, to meet the growing demand for innovative design talents worldwide, the Taiwan government has been committed to promoting policies related to the cultivation of innovative and creative talents nowadays, particularly for innovative design profession students who often use the Internet and short videos for learning. Therefore, the study aims to use questionnaires to understand the habits and addiction of the innovative design profession students in using short videos, and to further investigate the relation of short video addiction to the students’ creative self-efficacy (CSE) and career interests. A total of 561 valid questionnaires were collected after eliminating invalid questionnaires and reliability analysis. Structural equation modeling and model validation were conducted afterwards. The results showed that short video addiction had a negative effect on CSE; CSE had a positive effect on career interests; and CSE had an indirect effect between short video addiction and career interests.

## 1. Introduction

According to the “2020 Communications Market Survey in Taiwan” and “2020 Taiwan Internet Report”, the use and access of the Internet of youths aged from 16 to 25 are the highest among all age groups: the Internet access rate is approaching 100% and the mobile Internet access reaching over 93%. Among the various Internet usage behaviors, instant message accounts for 14.2%, social media 13%, and audio-visual entertainment 11.1% respectively [1,2]. This shows that the mobile Internet has a high demand for youths’ audio-visual and social behaviors.

With regard to the COVID-19 pandemic in the past two years, the resources and demand for e-learning in schools and society has greatly increased. Numerous emerging online media, such as TikTok, Kuaishou, YouTube and other audio-visual platforms are constantly appearing and being used in learning, teaching aids and life, which has a great impact on the living habits, learning styles and values of youths. As a result, it seems to be an indispensable learning channel for teenagers to use short video related application on smartphones to obtain knowledge and skills. Relevant studies have also demonstrated that the smartphones can help students not only learn anywhere but also improve peer interaction, maintain interpersonal relationship, improve learning tactics, plan after-school learning, document learning processes and write assignment reports [3,4,5]. However, numerous studies have also pointed out that college students are not only the main users of short videos, but also have a high risk of short video addiction [6]. More specifically, short video addiction refers to the excessive use of short videos that leads to dysfunctional physical, psychological and social function, resulting in negative emotion, low self-esteem, lack of happiness and sleep difficulties [7,8,9,10,11]. In short, it is clear that short video addiction still has many negative effects on college students. These studies have mainly investigated the physiological and psychological influences of short video addiction on students, and less attention has been paid attention to the learning process and future development of students in the professional fields.

From the above-mentioned studies, it has been proven that the popularity of short videos would cause the risk of addiction to college students [12,13]. More research has tended to focus on the relationship between addiction and creativity [14,15,16]. Particularly, for students in innovative design profession, creativity is not merely the key to competence development; their learning style and learning content largely rely on the use of the Internet, so as to gather ideas from short videos or gain inspiration from other pieces. Compared to students in other fields, innovative design profession students are more susceptible to the effects of short video addiction. Related studies have showed a positive correction between the innovative design profession and creative self-efficacy (CSE) [17], indicating that students with innovative design background should have higher CSE performance.

From the viewpoint of self-efficacy, it is seen as a belief in one’s own abilities [18]. People with high self-efficacy are able to increase their personal and overall achievement and benefit; to take challenges as tasks rather than treats to be avoided, and to ensure that they can master the difficulties. Conversely, people with low self-efficacy doubt their abilities while facing difficult tasks, first considering obstacles and their own weaknesses, and detailing various possible negative outcomes rather than thinking about ways to overcome. Self-efficacy theory can be applied in a number of domains, and many studies have demonstrated its application to CSE [19,20,21].

CSE is defined as “the belief one has the ability to produce creative outcomes” [22]. Social psychologist, Amabile [23] stated that CSE is influenced by professional knowledge, creativity and career interests. Professional knowledge is the basis of creativity. Without a certain level of professional knowledge, it is difficult to have a good creative performance [24,25]. Then, creativity is a method and technique that enhance creative thinking and CSE [26,27]. Additionally, career interests are a key factor in the performance of CSE. The higher the interest in work, the better the CSE will be [28,29]. In brief, short video addiction, CSE and career interests are all receiving much attention in the education field, but there is a lack of studies examining the association among them. Thus, the study is examined by self-administered questionnaire (see Appendix A) and aims to investigate the impact of short video addiction on the interaction of creative self-efficacy and career interests in innovative design profession students.

## 2. Methods

### 2.1. Model and Hypothesis

#### 2.1.1. Self-Efficacy

According to Bandura’s [18] self-efficacy theory, self-efficacy is an individual’s belief that he or she has sufficient ability for accomplishment. Self-efficacy is not related to the skill an individual possesses, but rather to the self-judgment of the degree of ability possessed, as self-efficacy affects how an individual tries to achieve something or how much effort he or she puts into completing it [30]. With regard to several studies, there is a tight correlation between self-efficacy, expertise identification, and learning engagement. The more students identify with their expertise, the more willing they are to work hard [31,32,33]. Therefore, self-efficacy theory has been widely applied in various fields, including creative self-efficacy [19,20,21]. CSE can be seen as an indicator to assess the performance and development of one’s creativity [34,35], as well as the belief in the performance of one’s own creativity [36], in which CSE plays a crucial role in innovative design profession students.

#### 2.1.2. Research Model

**Figure 1:**
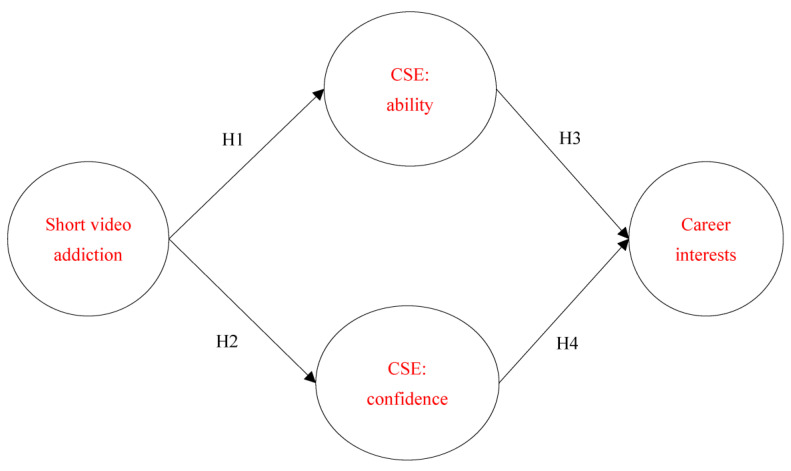
Research Model.

According to Hong [37] work on CSE, which categorized high school students’ CSE into confidence and problem-solving ability, the study takes a step further and investigates the effects of short video addiction on CSE and career interests of college students in the innovative design profession. The research model is shown as Figure 1.

#### 2.1.3. Hypothesis

1. Short Video Addiction and CSE

In the past few years, more and more studies have been conducted to investigate the relations between creativity and creative self-efficacy. Most of the studies have shown a positive correlation between creativity and creative self-efficacy [17,38,39], which exposes that students with innovative design profession might outperform students in other fields in creative self-efficacy. Additionally, relevant studies have shown that addictive behaviors have impacted on students’ self-efficacy, learning motivation, and learning achievement [12,40,41,42,43,44,45,46,47,48]. Thus, the study proposed the following hypotheses:

**Hypothesis 1 (H1)**  *Short video addiction has a negative effect on CSE-ability*.

**Hypothesis 2 (H2)**  *Short video addiction has a negative effect on CSE-confidence*.

2. CSE and Career Interests

Several studies have demonstrated that the level of self-efficacy is highly correlated with learning motivation, and students with high self-efficacy tend to have higher learning motivation [49,50,51,52,53,54,55]. Meanwhile, not only can learning motivation further influence students’ career interests [56,57,58,59,60], but several studies also pointed out that CSE can be seen as a factor to affect or predict students’ career development [28,61,62]. Thus, the study proposed the following hypotheses:

**Hypothesis 3 (H3)**  *CSE-ability has a positive effect on career interests*.

**Hypothesis 4 (H4)**  *CSE-confidence has a positive effect on career interests*.

3. Short Video Addiction, CSE and Career Interests

From the above-mentioned studies, an interactive relationship among short video addiction, creative self-efficacy, and career interests is observed. Therefore, to understand the relation between each dimension, the hypotheses are conducted as follows:

**Hypothesis 5 (H5)**  *CSE has an indirect effect on short video addiction and career interests*.

### 2.2. Procedure

The current study was an empirical study and recruited college students in innovative design profession as the participants. Snowball sampling method was applied. The questionnaire link was sent to a number of innovative design-relevant departments to invite students to fill it out. The questionnaire was collected since 1 March 2022, and the collecting process was completed once 650 copies of the questionnaire were received. It was ensured that all processes conducted in the study comprising humans were in accordance with the ethical principles of the American Psychological Association. Informed consent was provided in the questionnaire, so that all participants were aware that they were participating in this study, and that the data they provided were presented anonymously.

### 2.3. Participants

The current study recruited 650 participants (the number of returned questionnaires). The total number of invalid pieces was 89, while the number of the valid pieces (participants) was 561, with a valid return rate of 86.3%. Among 561 study participants, there were 247 male students (44%) and 314 female students (56%); 175 first-year students (31.2%), 112 second-year students (20%), 183 third-year students (32.6%), and 91 fourth-year students (16.2%). With regard to the average number of weekly viewers, there were 75 participants viewing for 1–3 days (13.4%), 124 participants viewing for 4–6 days (22.1%), and 362 participants viewing daily (64.5%). Additionally, regarding the average number of daily viewers, there were 58 participants watching less than 1 h (10.3%), 279 participants watching between 1-3 h (49.7%), 201 participants watching between 3-5 h (35.8%), and 23 participants watching for more than 5 h (4.1%). The average age of participants was 19–20 years old, with a standard deviation of 1.49 years.

### 2.4. Instruments

#### 2.4.1. Short Video Addiction

Short video addiction is seen as a psychiatric disorder and a complex bio-psychological phenomenon. It is defined as the inability of users to control their media use, resulting in disruption of their daily life. From the definition, the current study refers to the Short Video Addiction Scale by Ye [12] and Liu [13], which measures the participants’ perceived level of self-viewing short video addiction by 10 questions. For example, “I drop what I should be doing and spend my time watching short videos” and “I get angry if someone interrupts me while I am watching short videos”.

#### 2.4.2. CSE

The current study refers to the Creative Self-Efficacy Scale of Hong [36] with seven questions to explore the ability and confidence of creative self-efficacy to students in innovative design profession. For example, “I am confident that I can solve problems in many new approaches” and “I am always confident to solve the design problems of my work”.

#### 2.4.3. Career Interests

According to the study of Cui [63], the experience of creative invention mediated career interests. The current study applied Cui’s [36] study instrument, Career Interest Questionnaire, to explore the association between students’ CSE and career interests through seven questions. For example, “In the future, I would like to find a job with challenge to solve problems” and “In the future, I would like to find a job related to innovation”.

### 2.5. Data Analysis

Structural equation modeling (SEM) is a statistical modeling technique that can be used to test complex causal structure [64]. To ensure the reliability of the questionnaires of the study, the items of questionnaires were conducted for pretest, item analysis, reliability and validity analysis, and model overall fit measures. Formal questionnaire implementation was conducted after taking out invalid question items, and model validation and indirect effect analysis was applied to the results afterward.

## 3. Results

### 3.1. Item Analysis

To ensure the questionnaire was consistent and stable, item analysis was applied initially. Relevant studies pointed out it was adequate that χ^2^/df value was between 1–3, RMSEA value was below 0.10 and GFI value was above 0.90 [65,66,67]. According to the results of the item analysis, the question items of short video addiction were decreased from 10 to 6 questions, CSE-ability was from 7 to 5 questions, CSE-confidence was from 7 to 6 questions, and career interests was from 7 to 5 questions. As Table 1 presents below, the results of the confirmatory factor analysis show that the dimensions of short video addiction, CSE-ability, CSE-confidence and career interests all met the criteria for goodness of fit.

### 3.2. Reliability and Validity Analysis

Some related studies highly suggested that the reliability and validity should be above 0.70 [68,69]. According to the results of the item analysis, the alpha value of each dimension was achieved as >0.70, which represents the reliability of stability and consistency among the question items of the dimensions of short video addiction, CSE-ability, CSE-confidence and career interests.

The CR values of each dimension reached >0.70, indicating that there was consistency in the latent variables among dimensions of short video addiction, CSE-ability, CSE-confidence and career interests. The six questions of the dimension of short video addiction had high internal consistency, indicating that these six questions were sufficiently representative of the dimension of short video addiction.

Additionally, the AVE of each dimension reached >0.50, demonstrating that the four dimensions were distinguished from each other, which each of the four dimensions being independent (discriminant validity test). In brief, the short video addiction, CSE-ability, CSE-confidence and career interests are sufficiently distinguished with stability and consistent reliability, as shown in Table 2.

### 3.3. Model Overall Fit Measures

Structural equation modeling (SEM) has become a standard tool in many scientific fields for explaining the interrelationships between variables and the soundness of theoretical models [70]. It is noted that GFI value achieved at 0.8 and above, which shows the goodness of fit of the model [70,71]. The fitted index values for the study were χ^2^ = 919.5, df = 205, χ^2^/df = 4.49, RMSEA = 0.08, GFI = 0.89, AGFI = 0.87, NFI = 0.89, NNFI = 0.90, CFI = 0.91, IFI = 0.91, RFI = 0.88, PNFI = 0.79 and PGFI = 0.72.

### 3.4. Model Validation

**Figure 2:**
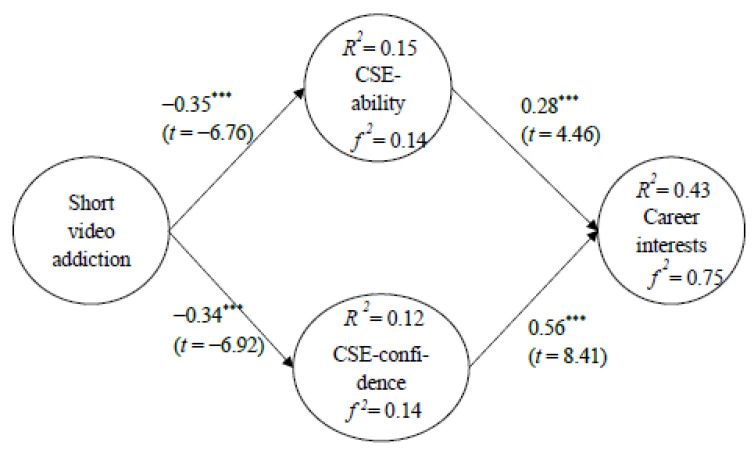
Validation of research model. *** *p* < 0.001.

The current study proposed five research hypotheses and constructed a theoretical model, which was validated by SEM. The results of the validated model showed that short video addiction had a negative effect on CSE-ability (γ 1 = −0.035, *p* < 0.001); short video addiction had a negative effect on CSE-confidence (γ 2 = −0.034, *p* < 0.001); CSE-ability had a positive effect on career interests (γ 3 = 0.028, *p* < 0.001); CSE-confidence had a positive effect on career interests (γ 4 = 0.056, *p* < 0.001); and CSE played a medicating role in short video addiction and career interests. As shown in Figure 2, the explanatory power of short video addiction on CSE-ability was 15%, and f 2 was 0.14; the explanatory power of short video addiction on CSE-confidence was 12%, and f 2 was 0.14; the explanatory power of CSE-ability and CSE-confidence on career interests was 43%, and f 2 was 0.75.

### 3.5. Indirect Effect Analysis

Table 3 illustrates the results of indirect effect analysis. It showed that short video addiction had an indirect effect on career interests (β = −0.29 **).

## 4. Discussion

Short video addiction is a psychiatric disorder and a complex bio-psychosocial phenomenon. It is defined as the addictive behavior of users who excessively, inappropriately or dependently use short video software [12,72]. Short video addiction is a kind of behavior that takes smart phones and short video media as a tool and has poor self-control, resulting in the constant need of short video watching. The main manifestations of short video addiction include short video watching for long periods of time with self-condemnation, being conscious of the need to control the short video watching while not being able to control the behavior of short video watching [73,74]. With regard to the results analysis, the participants of the current study had a low level of short video addiction (M = 2.36, SD = 0.81). CSE refers to an individual’s beliefs and ability to expect themselves to produce creative outcomes [22]. CSE-ability of the participants in the current study was moderate (M = 3.25, SD = 0.67) and CSE-confidence was moderate (M = 3.21, SD = 0.68). Career interests were exploring students’ perceptions of choosing a career path that is aligned with their professional abilities [63,75,76], and the participants of this study had a moderate level of career interests (M = 3.29, SD = 0.70).

From the above results, Kassa and Palma’s [77] point of view could explain the low overall mean score of short video addiction, which the participants were investigated by self-assessed questionnaire, and it might be affected by social values. However, the current study does not aim at descriptive analysis, but a correlation study of each dimension in the model. From the validation results, it is proven that the hypothetical paths of each dimension are still valid.

### 4.1. Short Video Addiction Has a Negative Impact on Creative Self-Efficacy

The results of the current study suggest that short video addiction has negative effects on both CSE-ability and CSE-confidence. The results are consistent with most of the relevant studies which show that the addiction has more negative than positive effects on emotional and psychological problems [12,78,79]; it also leads to pessimistic learning motivation [12,40,41,42,43,44,45,46,47]. It indicates that high levels of short video addiction would negatively influence students’ CSE. In short, higher levels of short video addiction might reduce students’ ability and confidence in creative development.

### 4.2. CSE Has a Positive Effect on Career Interests

The findings of the current study uncover that both CSE-ability and CSE-confidence have positive effects on career interests, indicating that CSE, professional knowledge, and career interests would affect each other, which is consistent with a number of prior studies [23,28,61,62,63]. In brief, the higher the students’ CSE is, the better choice of jobs in line with their profession is.

### 4.3. CSE has an Indirect Effect on Short Video Addiction and Career Interests

The results of the current study suggest that CSE mediates an indirect effect between short video addiction and career interests. According to previous studies, short video addiction would affect CSE, and CSE would also affect career interests. Therefore, the results are anticipated and consistent with related studies which reveal that the excessive use of social media has a negative effect on the physical and mental health of youths [7,8,9,10,11,80].

## 5. Conclusions and Suggestions

### 5.1. Conclusions

The aim of the study was to understand the effects of short video addiction on CSE and career interests of innovative design profession students, and also to further develop a research model to investigate the relationship between short video addiction, CSE and career interests. The results showed that (1) short video addiction has a negative effect both on CSE-ability and CSE-confidence; (2) both CSE-ability and CSE-confidence have a positive effect on career interests; (3) CSE plays a mediating role between short video addiction and career interests.

In addition, although the level of short video addictive behavior of the participants examined was low in the study, some relevant studies presented that most students still have had the frequent and prolonged short video watching behavior [1,2]. The cause of the consequence might be due to the influence of social value or cognitive gap, in that the students were not aware it was an addiction behavior. However, the focus of the current study aims to demonstrate that short video addiction would affect the CSE and the development of career interests in innovative design profession students. Thus, the more important issue is how to prevent short video addiction from weakening the students’ CSE and the development of career interests.

### 5.2. Suggestions

With regard to the results of the current study, short video addiction was proven to have a negative impact on students’ CSE and career interests. Yet, due to the influence of popularity of short video, it is not feasible to thoroughly eradicate the use of short videos. Short video watching is not merely inadequate for students, but if short videos for learning are effectively used, they can enhance students’ willingness and effectiveness in learning [81,82,83,84]. Accordingly, parents and teachers should take the role to strengthen students’ self-regulated ability and progressively guide them to control the use of short video applications, or to provide short videos that can assist professional learning. By doing this, the engagement of short video can avoid affecting students’ CSE development and further accelerate their interests in learning and professional development.

### 5.3. Limitations and Works for Future Research

The study was conducted by quantitative analysis, and the corresponding results and conclusions were inferred by the statistical examination. Although the correlation between short video addiction and CSE was confirmed, it was insufficient to understand the process and effect of how the student watched short videos. As a result, by adopting qualitative interviews to gain profound understanding of the participants’ psychological states and actual thoughts, the results of the study can alternatively have more referred value.

Based on the study, although short video addiction has a negative impact on innovative design profession students, it is necessary to observe that students are so accustomed to watching short videos nowadays. Therefore, it is suggested that future research can explore the use of short videos for learning in specific professional fields, thus transforming short videos into one of the learning tools and further exploring the impact on students’ learning effectiveness.

Finally, and above all, the study focuses on the effects of short video addiction on CSE and career interests. However, short video addiction is only one of the Internet addictive behaviors. It is suggested that a comparative study can be conducted to investigate the effects of different Internet addictive behaviors on CSE or career interests in the future to enrich the relevant topics within CSE research.

## Figures and Tables

**Table 1:** Confirmatory factor analysis.

Goodness of Fit	Critical Value	Short Video Addiction	CSE- Ability	CSE- Confidence	Career Interest
χ^2^	---	22.9	10.4	17.8	22.7
df	---	9	5	9	5
χ^2^/df	<5	2.54	2.08	1.98	4.54
RMSEA	<0.10	0.05	0.04	0.04	0.08
GFI	>0.80	0.99	0.99	0.99	0.99
AGFI	>0.80	0.97	0.98	0.98	0.96
FL	>0.50	0.64~0.86	0.63~0.87	0.76~0.89	0.66~0.89
t	>3	17.84~27.53	18.47~22.90	19.04~23.13	17.63~24.54

**Table 2:** Reliability and validity analysis.

Construct	M	SD	α	CR	AVE	FL
	---	---	>0.70	>0.70	>0.50	>0.50
Short video addiction	2.36	0.81	0.87	0.87	0.54	0.73
CSE-ability	3.25	0.67	0.89	0.90	0.65	0.80
CSE-confidence	3.21	0.68	0.92	0.92	0.67	0.82
Career interests	3.29	0.70	0.89	0.88	0.60	0.77

**Table 3:** Indirect Effect Analysis.

Dimension	Short Video Addiction	
	β	95% CI
Career interests	−0.29 **	[−0.38, −0.17]

** *p* < 0.01.

## Data Availability

The original contributions presented in the study are included in the article; further inquiries can be directed to the corresponding author.

## Appendix A

1.Gender	□Male □Female
2.Age	_______years old
3.Grade	□freshman □sophomore □junior □senior
4.Number of days per week for short video watch	□1–3 days □4–6 days □7 days
5.Per time for each short video watching time	□less than one hour □one-three hours□three-five hours □more than five hours
6.Preferring short video type	□existentialism □special talent □cosmetics/costume □facial attractiveness □daily documentary □comedy □street interview □animation □delicacy □popular science □parenting/childcare □budding pet
7.The most frequent short video platform you’ve watched	□Tik Tok □Kuaishou □Huoshan short video □others_____

Please Check at the Appropriate ☐ for the Following Questions Regarding your Intuition. There is no Need to Think Long about the Answer, and Do not Miss Any Questions.		**Strongly Disagree**	**Disagree**	**Neutral**	**Agree**	**Strongly Agree**
**Short Video Addiction (SVA)**						
1.	I’ll spend more time watching the short videos than I originally expect.	1☐	2☐	3☐	4☐	5☐
2.	I’ll let go of what I need to accomplish and spend my time watching short videos.	1☐	2☐	3☐	4☐	5☐
3.	My excitement and anticipation of watching short videos is much higher than other interactions.	1☐	2☐	3☐	4☐	5☐
4.	I’ll be complained or blamed by others for watching short videos.	1☐	2☐	3☐	4☐	5☐
5.	I’ll be late to school, leave early, or be absent because of watching short videos.	1☐	2☐	3☐	4☐	5☐
6.	My study will be regressed because of watching short videos.	1☐	2☐	3☐	4☐	5☐
7.	I get mad if someone interrupts me while I’m watching a short videos.	1☐	2☐	3☐	4☐	5☐
8.	I’ll sacrifice the sleep at night by watching short videos.	1☐	2☐	3☐	4☐	5☐
9.	I’ll not forget the content of the short videos even after I turn it off.	1☐	2☐	3☐	4☐	5☐
10.	I’ll be depressed because I don’t watch the short videos.	1☐	2☐	3☐	4☐	5☐
**Creative self-efficacy (CSE): ability**						
1.	I feel I have the ability to come up with many innovative ideas.	1☐	2☐	3☐	4☐	5☐
2.	I’m very good at finding creative ways to solve problems.	1☐	2☐	3☐	4☐	5☐
3.	When I encounter a problem in design (working on a project), I can always get to the root of the problem.	1☐	2☐	3☐	4☐	5☐
4.	When I encounter a trouble in design (working on a project), I always analyze that troublesome situation.	1☐	2☐	3☐	4☐	5☐
5.	When I encounter a problem in design (working on a project), I can always generate new ideas to solve the problem.	1☐	2☐	3☐	4☐	5☐
6.	When I encounter an unrealistic expectation in design (working on a project), I can always adjust my design plan.	1☐	2☐	3☐	4☐	5☐
7.	When I encounter an inappropriate methodology in design (working on a project), I can always assess the consequences of changing the method or technique.	1☐	2☐	3☐	4☐	5☐
**Creative self-efficacy (CSE): confidence**						
1.	I always have the confidence to challenge new problems.	1☐	2☐	3☐	4☐	5☐
2.	I’m confident that I can solve problems in many new ways.	1☐	2☐	3☐	4☐	5☐
3.	I believe the ideas I come up with are very creative.	1☐	2☐	3☐	4☐	5☐
4.	I always have the confidence to deal with problems in design (working on a project).	1☐	2☐	3☐	4☐	5☐
5.	I always have the confidence to quietly figure out how to solve the design problems (working on a project).	1☐	2☐	3☐	4☐	5☐
6.	I’m always confident that I can solve the design problems (working on a project).	1☐	2☐	3☐	4☐	5☐
7.	When I encounter an inappropriate method in design (working on a project), I’m always confident that I can evaluate the consequence of changing the method or technique.	1☐	2☐	3☐	4☐	5☐	
**Career interests**						
1.	I’d like to find a job where I can be challenged to solve problems in the future.	1☐	2☐	3☐	4☐	5☐
2.	I’d like to find a job where I can continue to invent and create in the future.	1☐	2☐	3☐	4☐	5☐
3.	I’d like to find a job where I can add new features to my work piece in the future.	1☐	2☐	3☐	4☐	5☐
4.	I’d like to find a job where I can develop new work piece in the future.	1☐	2☐	3☐	4☐	5☐
5.	I’d like to find a job that can improve human life in the future.	1☐	2☐	3☐	4☐	5☐
6.	I’d like to find a job related to innovation in the future.	1☐	2☐	3☐	4☐	5☐
7.	I’d like to find a job where I can discover knowledge in the future.	1☐	2☐	3☐	4☐	5☐
